# Genetic prescreening of a candidate for laser refractive surgery identifies risk for inadequate tissue response: a case report 

**DOI:** 10.1186/s13256-022-03395-7

**Published:** 2022-05-16

**Authors:** Andrea Cusumano, Hila Roshanravan, Connie Chao-Shern, Jacopo Sebastiani, Jung Hee Levialdi Ghiron, Larry DeDionisio, Tara Moore

**Affiliations:** 1 University of Rome Tor Vergata, Rome, Italy; 2 Avellino Labs, Menlo Park, CA USA; 3 Macula & Genoma Foundation Onlus, Rome, Italy; 4 Ulster University, Coleraine, UK

**Keywords:** Refractive surgery, Corneal ectatic disease, Genetic testing

## Abstract

**Background:**

Inadequate response to corneal laser refractive surgery, e.g., ectatic corneal diseases, may not be identified by conventional examinations, hence creating therapeutic uncertainty. Herein we demonstrate the application of genetic prescreening to augment preassessment for corneal laser refractive surgery and highlight the ability to prevent the possibility of enrolling a subject at risk for developing ectatic corneal diseases.

**Case presentation:**

Preoperative tests were performed alongside deoxyribonucleic acid (DNA) sequencing of 75 genes specific to the structure and health of the eye of a 44-year-old Caucasian male candidate for corneal laser refractive surgery. The patient had no medical, family, or psychosocial history, nor symptoms that could lead to suspect any corneal abnormalities, and conventional preoperative tests confirmed that no corneal abnormalities were present. The sequencing results uncovered rare DNA variants within the *ADGRV1*, *PTK2*, *ZNF469*, and *KRT15* genes. These variants were considered potential risk factors for inadequate response in the patient post corneal laser refractive surgery. Subsequent reevaluation with three different last-generation corneal tomographers identified in the left eye a “warning” for a deformity of the posterior profile of the cornea.

**Conclusions:**

Genetic prescreening identifies potential risk of inadequate response to corneal laser refractive surgery where current technologies in use may lead to a hazardous predictive diagnostic uncertainty.

## Background

The possibility of postoperative ectasia following corneal laser refractive surgery (CLRS) is an ongoing concern within the ophthalmic community [1, 2]. Although uncommon, occurrences of ectasia have been observed after laser-assisted *in situ* keratomileusis (LASIK) and have also been reported after less disruptive procedures such as small incision lenticule extraction (SMILE) [3, 4]. A predisposition to develop an ectatic corneal disease (ECD) such as keratoconus (KC) can lead to inadequate responses to CLRS. During the preoperative phase for patients considering CLRS, conventional examinations, including corneal topography, may not easily identify the propensity to develop an ECD.

Important information can be obtained regarding a patient’s risk for developing an ECD through genetic testing. Genetic factors are known to play a role in the etiology of ECDs such as KC [5, 6], and a wide range of evidence exists indicating a link between central corneal thickness (CCT), a highly heritable trait associated with ECD, and multiple loci within the human genome [7–11]. Thus, augmenting conventional examinations with a genetic prescreening (GP) test provides a powerful tool in determining risk for postoperative ectasia. Herein, we present a representative case illustrating the utility of GP in uncovering genetic variants within the deoxyribonucleic acid (DNA) of the patient and his children. We advocate GP to assess a patient’s candidacy for CLRS, thus preventing the possibility of enrolling subjects at risk of developing ECDs or inadequate responses to CLRS with poor postoperative visual outcomes and complex corneal healing.

## Case presentation

### Initial clinical examinations and genetic testing

**Fig. 1:**
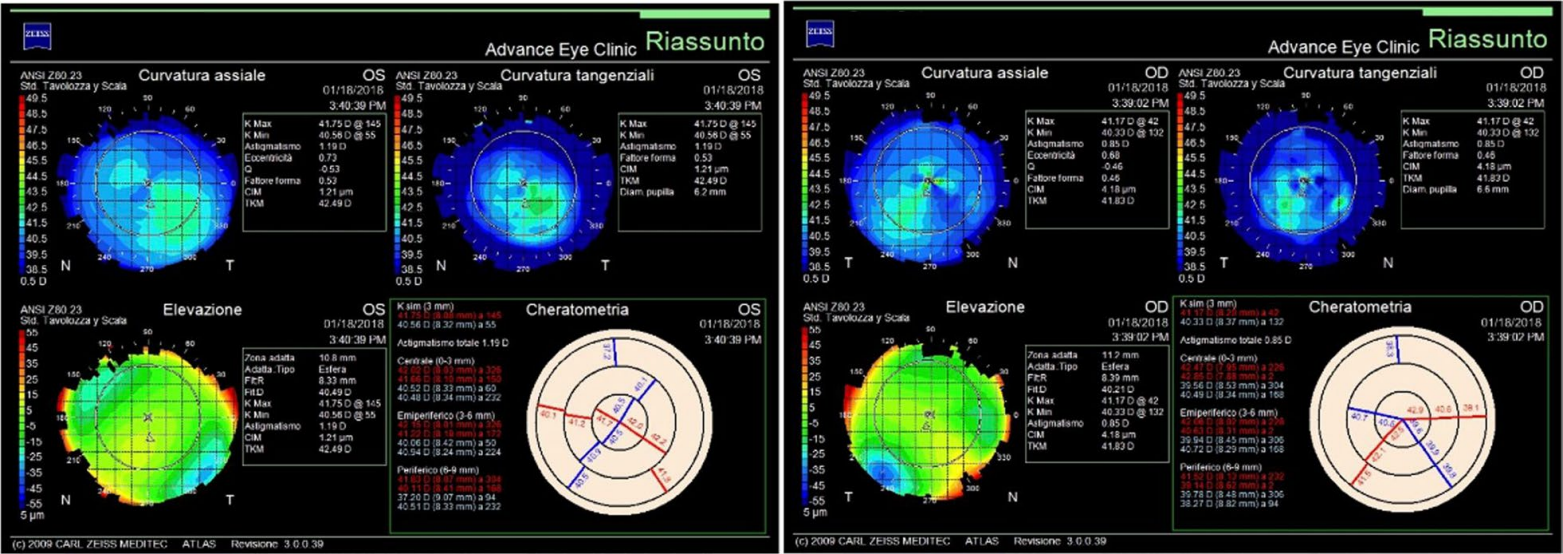
Topography map for both eyes of a patient considering corneal laser refractive surgery captured with a Zeiss ATLAS 9000

Preoperative tests, including corneal topography utilizing a Zeiss ATLAS 9000 (Carl Zeiss AG, Oberkochen, Germany), were performed on a 44-year-old Caucasian male patient considering CLRS. The patient did not have medical, family, or psychosocial history that could indicate predisposition or existence of corneal abnormalities, nor did he show any symptoms that could suggest corneal aberrations. Moreover, the patient had never undergone previous eye interventions. No financial, language, or cultural challenges occurred regarding the management of this case. The results from the initial presentation (T = 0) (that is, topographic examination) did not indicate corneal abnormalities in either eye of the patient (Fig 1), nor any other ocular pathologies. For GP, a buccal mucosa sample was collected by using an iSWAB collection kit (Mawi DNA Technologies, Hayward, CA, USA). After DNA was extracted (QIAamp DNA mini kit, QIAGEN Inc. Hilden, Germany) from the sample, a next-generation sequencing (NGS) test (AvaGen, Avellino Lab, Menlo Park, CA, USA) targeting 75 genes specific to the structure and health of the eye was used to analyze the patient’s DNA. The sequencing results identified four potentially pathogenic variants located on chr 5 (*ADGRV1*), chr 8 (*PTK2*), chr 16 (*ZNF469*), and chr 17 (*KRT15*) within the patient’s genome (Table 1). These DNA mutations were found to carry a risk factor for ECD based on the following analysis.

**Table 1:** Variants found in the patient’s and his children’s deoxyribonucleic acid

	Chr	Position	Ref. allele	Allele 1	Allele 2	Gene	dbSNP	Protein/amino acid	PolyPhen2 prediction HumDiv	LRT prediction	PROVEAN prediction
Father	5	90079091	A	A	G	*ADGRV1*	rs182698253	p.H4461R	Probably damaging	Deleterious	Damaging
	8	141771274	T	T	C	*PTK2*	N/A	p.Y526C	N/A	N/A	N/A
	16	88504716	G	G	T	*ZNF469*	rs568704426	p.G3585V	Benign	N/A	Neutral
	17	39674737	G	G	A	*KRT15*	rs201164162	p.R115C	Probably damaging	Deleterious	Damaging
Daughter	17	39674737	G	G	A	*KRT15*	rs201164162	p.R115C	Probably damaging	Deleterious	Damaging
Son	5	90079091	A	A	G	*ADGRV1*	rs182698253	p.H4461R	Probably damaging	Deleterious	Damaging
	16	88504716	G	G	T	*ZNF469*	rs568704426	p.G3585V	Benign	N/A	Neutral
	17	39674737	G	G	A	*KRT15*	rs201164162	p.R115C	Probably damaging	Deleterious	Damaging

### Sequence variant analysis

Adhering to the guidelines put forward by the American College of Medical Genetics and Genomics (ACMG) for the interpretation of sequence variants [12], the following criteria were applied to determine which variants within the patient’s test results were most likely damaging and thus carry a risk for ECD. Sequence variants were filtered based on their minor allele frequency (MAF) in the general population and protein coding changes, categorized as missense, STOP gain/loss, nonsense, or frameshift/non-frameshift indels. Variants with an MAF < 0.01 based on The Genome Aggregation Database (https://gnomad.broadinstitute.org/) were considered to be possibly pathogenic. The pathology for each variant was also gauged by using three in silico prediction tools: PolyPhen2-HDIV, LRT, and PROVEAN. Each tool aims to determine the likely impact on the transcribed amino acid sequence and translated protein due to a change in the DNA sequence. All genome positions were based on the Genome Reference Consortium Human genome build 37 (GRCh37.p13).

The filtering criteria resulted in the four heterozygous, missense mutations detailed in Table 1. The *ADGRV1* and *KRT15* variants were classified as possibly damaging, deleterious, and damaging by PolyPhen2-HDIV, LRT, and PROVEAN respectively, while the variants in the *PTK2* and *ZNF469* genes were classified as benign, neutral, or no data available by the in silico prediction tools we used.

### Gene network analysis

**Fig. 2:**
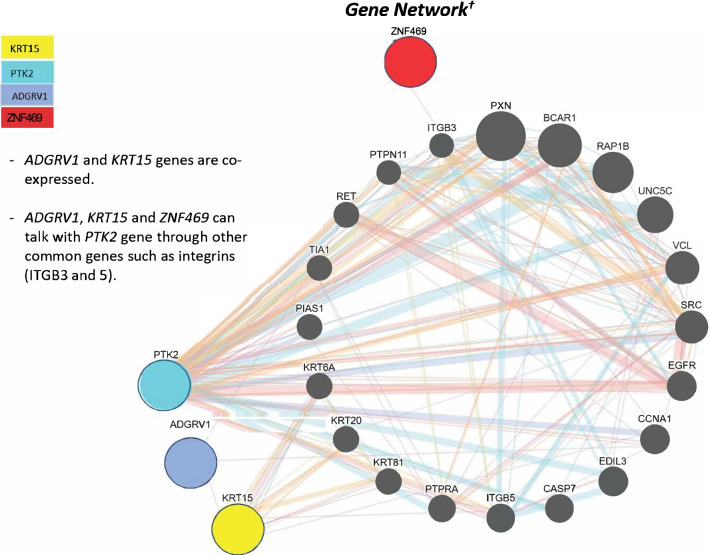
Gene network analysis utilizing the GeneMANIA server^†^. The analysis emphasizes the interactions of four genes and their impact on corneal metabolism. Within the DNA of a candidate for CLRS, four rare variants were detected in these genes via a NGS test assay^†^http://www.genemania.org

A further analysis was conducted by using the GeneMANIA server (http://www.genemania.org) [13] to provide insight of gene function for the four genes and how these variants within each gene could affect the health of the cornea via coordinated molecular pathways (Fig 2). This analysis found that *ADGRV1* and *KRT15* are coexpressed and that *ADGRV1*, *KRT15*, and *ZNF469* interact with *PTK2* via integrins, transmembrane cell adhesion receptors that play important roles in the regulation of cell migration during development, wound healing, and inflammatory response [14]. All four genes are integral to eye development and various molecular pathways. A few have been indicated in eye diseases of various types.

#### Adhesion G protein-coupled receptor V1 (*ADGRV1*) variant ID: rs182698253

The missense variant located in *ADGRV1* results in a change in the amino acid sequence of histidine to arginine at position 4661 (H4661R) within a region of the ADGRV1 protein that overlaps with a Calx-beta domain. The Calx-beta motif is a protein motif that is used for calcium binding and regulation, and this variant may lead to conformational changes in the protein that affects the binding of the ADGRV1 protein to its interacting partners by disrupting calcium binding sites. *ADGRV1* is part of the *USH2* complex, which plays an essential role in the development of hearing and vision via cell surface receptor signaling pathways [15]. A variant within this gene has previously been reported in two brothers suffering from KC [16].

#### Protein tyrosine kinase 2 (*PTK2*) variant: no rsID

Currently, there is no evidence that links *PTK2* to ECD. However, the *PTK2* variant found in the proband induces an amino acid change of tyrosine to cystine (Y726C) in the functional domain of the PTK2 protein. *PTK2* plays an essential role in regulating cell migration, adhesion, spreading, and reorganization of the actin cytoskeleton [17].

#### Zinc finger 469 (*ZNF469*) variant ID: rs568704426

This missense variant in the *ZNF469* gene results in the conversion of amino acid 3585 from glycine to valine (G3585V) within the ZNF469 protein. It is considered benign and neutral by PolyPhen2-HDIV and PROVEAN, respectively; however, the region where it is located overlaps with two regulatory regions that may affect binding of certain transcription factors (TFs) to their sites. There are numerous studies that have linked mutations in *ZNF469* to KC and to brittle cornea syndrome (BCS), an extreme form of ECD [18–22].

#### Keratin 15 (*KRT15*) variant ID: rs201164162

*KRT15* is expressed in corneal limbal epithelial cells and is involved in wound healing [23, 24]. The missense variant found in this gene, which results in an amino acid change from arginine to cysteine (R115C), overlaps with an intermediate filament rod domain in the protein structure. This plays a significant role during the protein assembly process, and mutations in this functionally important area disrupt end-to-end keratin interactions that could subsequently affect the corneal epithelia [25]. This variant also falls within a regulatory region that serves as a TF binding site [26]. Based on tissue and upstream signaling pathways, the related TF either enhances or suppresses the transcription of *KRT15*. Any dysregulation of *KRT15* at this TF binding site caused by this variant could affect changes in the cytoskeleton and could possibly affect the development of the lacrimal gland [26].

### Subsequent reevaluation with three different corneal tomographers

**Fig. 3:**
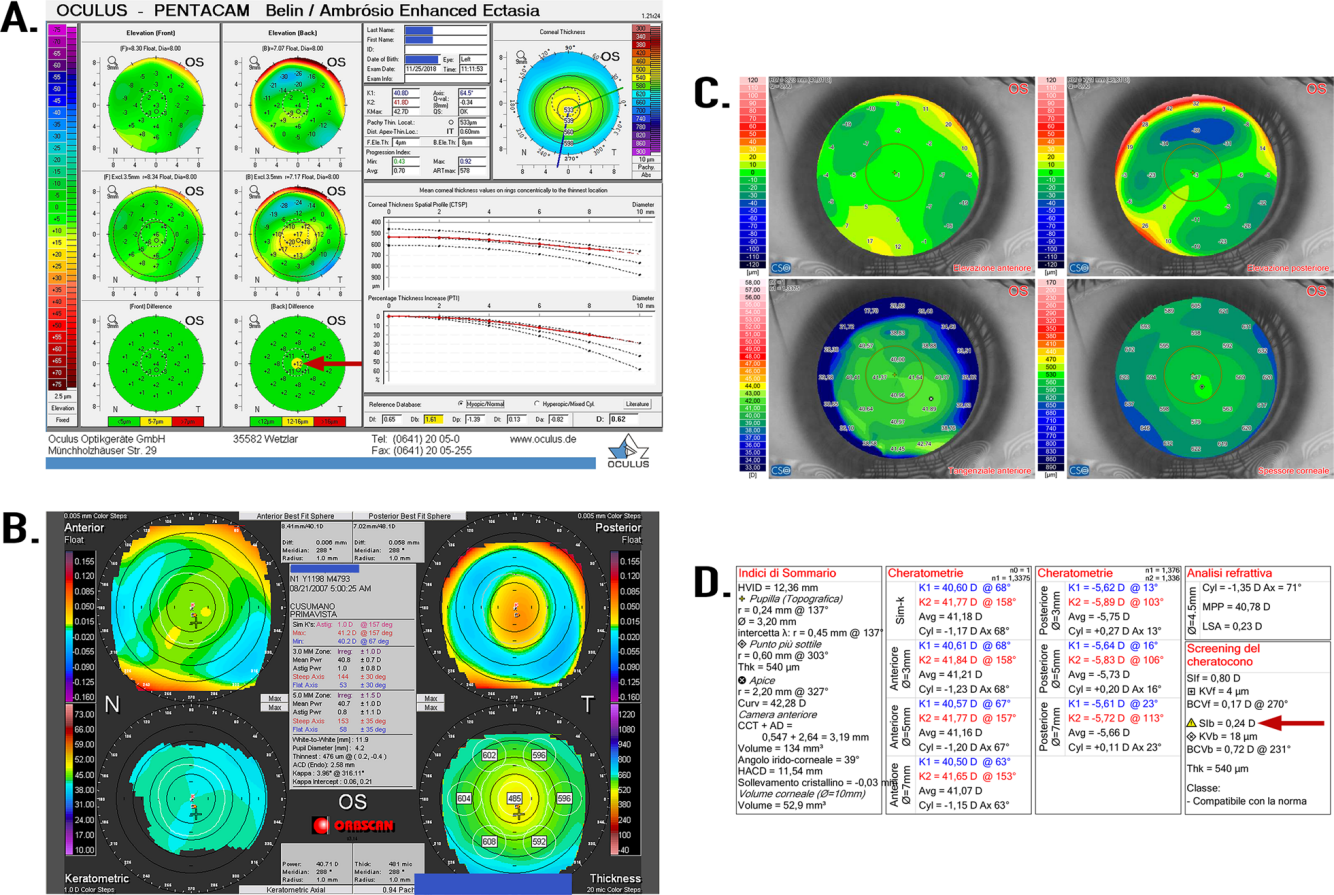
The difference in asymmetry between the anterior and posterior aspect of the cornea in the left eye (LE) illustrated by measurements obtained from the Pentacam, Orbscan, and SIRIUS instruments. The left eye was shown in all these tests to be “potentially” at risk. **A** The Pentacam examination was automatically evaluated with the Belin/Ambrósio method. The arrow points to a highlighted “warning” signal (yellow) in the rear differential map with a value of +12. **B** The Orbscan revealed the presence of an alteration of the corneal posterior elevation of 0.058 mm in the left eye. **C** The results of the SIRIUS show in the left eye the presence of an asymmetry of the curvature expressed in **D** (diopters) between the front and back face and which is represented with a yellow triangle, Slb = 0.24 D, indicated with an arrow.

After the results of GP determined the presence of potentially pathogenic variants, the patient was recalled and reevaluated with three different advanced tomographers (T = 52 days after initial presentation): a Pentacam (OCULUS, Wetzlar, Germany), an Orbscan (Bausch & Lomb, Rochester, NY, USA), and a SIRIUS (CSO, Florence, Italy). A deformity of the posterior profile of the cornea in the left eye (LE) was identified with all three instruments. The tomography measurements are shown in Fig. 3. The Pentacam produced a Belin/Ambrósio Enhanced Ectasia Display that highlighted a “warning” signal (yellow) that showed an elevation of 12 microns in the differential map of the back surface of the cornea (Fig 3A). The Orbscan showed the presence of an alteration of the corneal posterior elevation of 0.058 mm (Fig 3B). The results of the Sirius further confirmed the presence of an asymmetry of the curvature expressed in D (diopters) between the front and back face of the cornea in the LE, Slb = 0.24 D (Fig 3C).

## Discussion and conclusions

Genetic screening identified four rare variants in four genes within the DNA of a candidate for CLRS. Further clinical tests found an abnormality in the cornea of the patient’s LE. These results led to the decision to not perform any type of corneal laser refractive surgery due to the existing risk of inadequate responses in the patient’s cornea. Not having received any treatment, the patient was suggested to undergo follow-up visits every 12 months. Prior to GP, an initial corneal topography examination did not find any aberrations that would have warned the physician about possible poor postoperative results. DNA sequencing of the patient’s children uncovered, within the son, the variants located in the *ADGRV1*, *ZNF469*, and *KRT15* genes, while the daughter inherited the *KRT15* gene variant (Table 1). Based solely on the genetic inheritance pattern, the son is therefore at higher risk for developing an ECD than the daughter, since his genotype showed that he shared three of the four variants found in the father’s DNA. We must emphasize that the most common form of ECD, KC, is known to be a multifactorial disease [5, 6]. Environmental, behavioral, and genetic factors contribute to the onset of KC, and each of these factors should be considered in evaluating a risk for the disease.

### Evidence of a genetic risk for ECD

It is worth noting that the *KRT15* variant uncovered here could deleteriously affect other molecular pathways beyond the structure of the keratin 15 protein for which it encodes. Apart from its expression pattern in corneal limbal epithelial cells [23], the gene network analysis revealed that the region where this variant lies serves as a regulatory region crucial for lacrimal gland development [26]. We also note that *KRT15* is the host gene for *MIR6510*, a noncoding microRNA that which is involved in posttranscriptional regulation of gene expression and is part of the keratinization pathway. Further research is needed to fully understand how the R115C variant in *KRT15* might impact the functionality of *MIR6510*.

We determined that the *KRT15* gene is directly linked to *ADGRV1* through coexpression of their proteins. Given that the H4661R variant uncovered in *ADGRV1* could disrupt its functionality, we postulate that this variant, paired with the potential deleterious effects of *KRT15* variant, could ultimately alter the integrity of the cornea and play a role in the development of ECD. Furthermore, *KRT15* and *ADGRV1* indirectly interact with *ZNF469* and *PTK2* through cell surface transmembrane receptors know as integrins. Integrins recognize and bind to extracellular matrix (ECM) proteins. They comprise various α- and β-subunits, resulting in 24 different protein combinations that have overlapping substrate specificity and cell-type-specific expression patterns [27]. The gene network analysis (Fig. 2) indicated that *PTK2* activates the integrins *ITGB3* and *ITGB5*, and that *ZNF469* is part of an integrin complex with *ITGB3*. Since the Y726C variant discovered in *PTK2* could alter the structure of the encoded protein, it could affect its ability to activate its integrin substrates and subsequently alter interactions between the ADGRV1 and keratin proteins with the ECM.

In addition, *ZNF469* plays a role in collagen homeostasis and corneal structure. The ZNF469 protein expressed in the human cornea shares 30% homology with the helical regions of collagen I and collagen IV, suggesting that it may function as a transcription factor or extranuclear regulator factor for the synthesis or organization of collagen fibers [21, 28]. *ZNF469* may also be involved in the transforming growth factor (TGF)-β pathway [29], whose disturbance would lead to disarray of collagen in human cornea. During wound healing, TGF-β1 induces the expression of major ECM proteins such as fibronectin and collagen [30]. *ZNF469* has been linked to CCT and its association to KC [7, 9, 22] and is implicated in the etiology for BCS [20, 21]. Nonetheless, there is evidence suggesting that *ZNF469* is not causative for KC in some population groups of European descent [31, 32]. The evidence indicates that, when viewed in isolation of the other variants, the G3585V variant in *ZNF469* is most likely benign (Table 1); however, a combination of this variant in conjunction with the other damaging and deleterious variants in *KRT15* and *ADGRV1* most likely contributed to the phenotype illustrated in the patient’s corneal tomography (Fig. 3).

The gene network analysis (Fig. 2) also indicates that the *PTK2* variant may also participate in the morphologic phenotype. Overall, the evidence presented here points to the likelihood that corneal aberrations that could lead to ECD can be attributed to a combination of different variants within a patient’s genome, a composite of both benign and damaging, rare and common variants, a polygenic etiology. Given the various gene interactions among the four genes under scrutiny, we must not conclude that the patient’s son is at lower risk for ECD because the *PTK2* variant was not present in his DNA, nor can we state that the daughter is free of risk for ECD simply because she inherited only one potentially pathogenic variant from her father (R115C in *KRT15*).

The four variants identified in this report are in genes that are part of a molecular network crucial to a healthy cornea. Their predicted interdependency, and in the case of the *KRT15* and *ADGRV1* variants, potential pathogenicity, can affect the expected refractive correction and corneal healing after CLRS is performed. We demonstrate here how GP has the potential to identify risk of inadequate response to CLRS where even the most advanced technologies in use today may leave uncertainty for “borderline cases,” without being able to give a true indication of the potential risk for corneal ectasia.

## Data Availability

The datasets generated for this study are not publicly available; however, data are available from the corresponding author on reasonable request.

## Abbreviations

CLRS: Corneal laser refractive surgery
ECD: Ectatic corneal disease
GP: Genetic prescreening
KC: Keratoconus
